# Radiotherapy cannot prolong overall survival of young prostate cancer patients with bone metastases

**DOI:** 10.1186/s12967-016-0868-y

**Published:** 2016-04-27

**Authors:** Bo Peng, Cheng Yang, Jian He

**Affiliations:** Department of Radiation Oncology, Zhongshan Hospital, Fudan University, 180 Fenglin Road, Shanghai, 200032 China; Department of Plastic Surgery, Zhongshan Hospital, Fudan University, 180 Fenglin Road, Shanghai, 200032 China; Department of Urology, Zhongshan Hospital, Fudan University, 180 Fenglin Road, Shanghai, 200032 China; Shanghai Key Laboratory of Organ Transplantation, Shanghai, China

**Keywords:** Radiotherapy, Prostate cancer, Bone metastaese

## Abstract

**Background:**

Patients with prostate cancer is commonly diagnosed with bone metastases. With the growing use of prostate-specific antigen testing, the frequency of prostate cancer has progressively increased in patients younger than 70 years. Radiotherapy is recognized for its effect on local control of bone metastases, but whether it could prolong overall survival is still controversial.

**Methods:**

A total of 113 prostate cancer patients (<70y) with bone metastases were retrospectively analyzed. The Kaplan–Meier method was used for survival analysis with log-rank test. Multivariate analysis was performed to find the prognostic factors with the COX regression model.

**Results:**

The 1-, 2-, 3-, 5-, 7- and 10-year survival rates were 97.14, 82.86, 62.61, 38.76, 25.83 and 13.84 % respectively in the radiotherapy group, and 92.75, 73.91, 54.66, 36.63, 26.03 and 17.85 % respectively in the non-radiotherapy group, which showed no significant difference. Multivariate COX regression showed the overall survival was associated with alkaline phosphatase when bone metastases occurred and the number of bone metastases.

**Conclusion:**

With the advances in life-prolonging treatment of metastatic prostate cancer, radiotherapy may not be the first choice for young bone metastatic prostate cancer patients in order to improve survival.

## Background

Prostate cancer (PCa) is the most commonly diagnosed malignant tumor among males in developed countries and the second leading cause of cancer-related mortality [1]. Although PCa is usually considered as a disease of advanced age, its frequency has progressively increased in patients younger than 70 years with the growing use of prostate-specific antigen (PSA) testing [2]. Autopsy series on patients with PCa reveal that 80–85 % of them have bone metastasis, with the pelvis and the vertebrae being involved in nearly all cases [3]. Although PCa prefers to metastasize to skeleton, patients with PCa in whom bone metastasis develops have a relatively good survival prognosis, which may even be a few years. Therefore, local control of bone metastasis for patients with prostate cancer is more important than patients with other cancers.

Radiotherapy (RT) is considered as an effective treatment for local control of bone metastasis, which could reduce the skeletal-related events (SREs) [4, 5]. It is estimated that the mean cost for RT on bone metastases is as high as 7553 USD per episode in the United States, which has become a heavy burden for national health care system [6]. However, the long-term effect of RT on bone metastases is still controversial. In this retrospective study, we reviewed 113 young PCa patients (<70y) who had bone metastasis. Patients’ characteristics, treatment efficacy, and prognosis were analyzed. The result revealed that RT on bone metastases cannot prolong overall survival of young PCa patients.

## Patients and methods

### Patient collection

A total of 113 patients (<70y) with bone metastases from PCa who were treated between 1997 and 2012 at Zhongshan Hospital, Fudan University, were included. All of the patients were confirmed with primary PCa by pathological diagnosis and were diagnosed with bone metastasis through radioisotope scanning or magnetic resonance imaging.

### Collection of clinic pathological data

Patient age at diagnosis, Gleason score at initial diagnosis, treatment for primary prostate lesions, number of bone lesions, PSA and alkaline phosphatase (ALP) levels, organ metastases sites, regional and remote lymph node metastases, as well as follow-up duration and survival status, were retrospectively collected and reviewed. Serum PSA and ALP values were determined in the department of clinical laboratory of Zhongshan Hospital. The PSA values of the patients were characterized as 0–4 ng/ml, 4–20 ng/ml or more than 20 ng/ml, while the ALP values were characterized as less than 150 U/l, equal or more than 150 U/l. In the pathological examinations of the patients, Gleason scores were characterized as 2–4, 5–7 or 8–10. When determining the number of metastases by radioisotope scanning, the number of metastases in each vertebra and rib was calculated as one; in the statistical evaluation, the number of bone metastases was assessed as either single or multiple.

### Radiotherapy for bone metastases

Indications of RT for bone metastases included pain, risk of pathologic fracture, and neurologic complications arising from spinal cord compression and nerve root pain. For patients with multiple bone metastases, those with lesions causing pain or possibly spinal cord compression were first considered for RT. Bone metastatic status was recorded at the initial RT session for metastatic bone disease. Irradiation was delivered through a single posterior field or parallel opposed fields, depending on the location and depth of lesions according to CT or MRI. The majority of therapy was provided with 6-megavolt (MV) or 15-MV photons; however, electron therapy was also selected for those with shallow lesions such as in the ribs or skull, or extremity metastases. Radiation fields involved macroscopic tumor volume and 1 to 1.5 cm margins. In the case of vertebral bone metastases, radiation fields usually encompassed 1 normal vertebra above and below the metastatic lesions. If the lesions presented with soft-tissue extension concurrence, the radiation fields were enlarged on the basis of CT or MRI results. We scheduled the full radiation dosage at 46 Gy for the vertebral metastatic lesions and 50–60 Gy for soft-tissue concurrence beyond the spinal cord, in daily doses of 2 Gy/fraction, 5 times a week. However, factors that indicated the need for a reduced dose were considered, such as progressive primary disease, many lesions, poor Karnofsky performance status, adverse effects, and patient inconvenience during RT.

### Follow-up and statistical methods

There were 5 in the non-RT group and 4 patients in the RT group lost to follow-up. The survival time was defined as from the date the first diagnosis of bone metastases to the date of death or of the last follow-up. The univariate and multivariate analyses were performed using SPSS 18.0 software (IBM, Armonk, NY, USA). The Kaplan–Meier method with a log-rank test was used for survival rate calculations and to evaluate each variable. Multivariate analysis was carried out with the Cox regression model, and all of the variables were analyzed with the method “enter”. All of the tests were two-sided, and *p* < 0.05 was considered statistically significant.

## Results

### Patient characteristics

A total of 104 patients (92.0 %) were followed up until the date of death or 30 June 2015. The average follow-up time for all patients was 50.01 months (SD = 32.94) while the median was 41 months (range from 1 to 177 months). All of the patients received endocrine therapy, including surgical castration, androgen ablation, or maximal androgen blockade. Among them, 35 patients received radiotherapy on bone metastases and 69 patients did not. The age, Gleason score, PSA level, ALP level, rate of receiving chemotherapy, the number of bone metastases, or other organ metastasis showed no significant difference between the RT and non-RT group. Only the rate of regional node metastasis showed significant difference. The baseline characteristic data are shown in Table 1.

**Table 1:** Baseline characteristics of patients

	RT	Non-RT	*P* value
Age	64.26 ± 4.98	63.96 ± 5.48	0.788
Gleason score	7.91 ± 1.31	7.88 ± 1.33	0.913
PSA	323.05 ± 529.83	368.14 ± 613.40	0.713
ALP	304.32 ± 455.35	316.52 ± 346.32	0.884
Chemotherapy			0.162
Yes	9 (25.71 %)	10 (14.49 %)	
No	26 (74.29 %)	59 (85.51 %)	
The number of bone metastases			0.975
Single	6 (17.14 %)	12 (17.39 %)	
Multiple	29 (82.86 %)	57 (82.61 %)	
Regional lymph node metastasis			0.002
Yes	1 (2.86 %)	20 (28.99 %)	
No	34 (97.14 %)	49 (71.01 %)	
Other organ metastasis			0.09
Yes	7 (20.00 %)	25 (36.23 %)	
No	28 (80.00 %)	44 (63.77 %)	

*PSA* prostate-specific antigen; *ALP* alkaline phosphatase; *RT* radiotherapy

### Bone metastasis sites

**Fig. 1:**
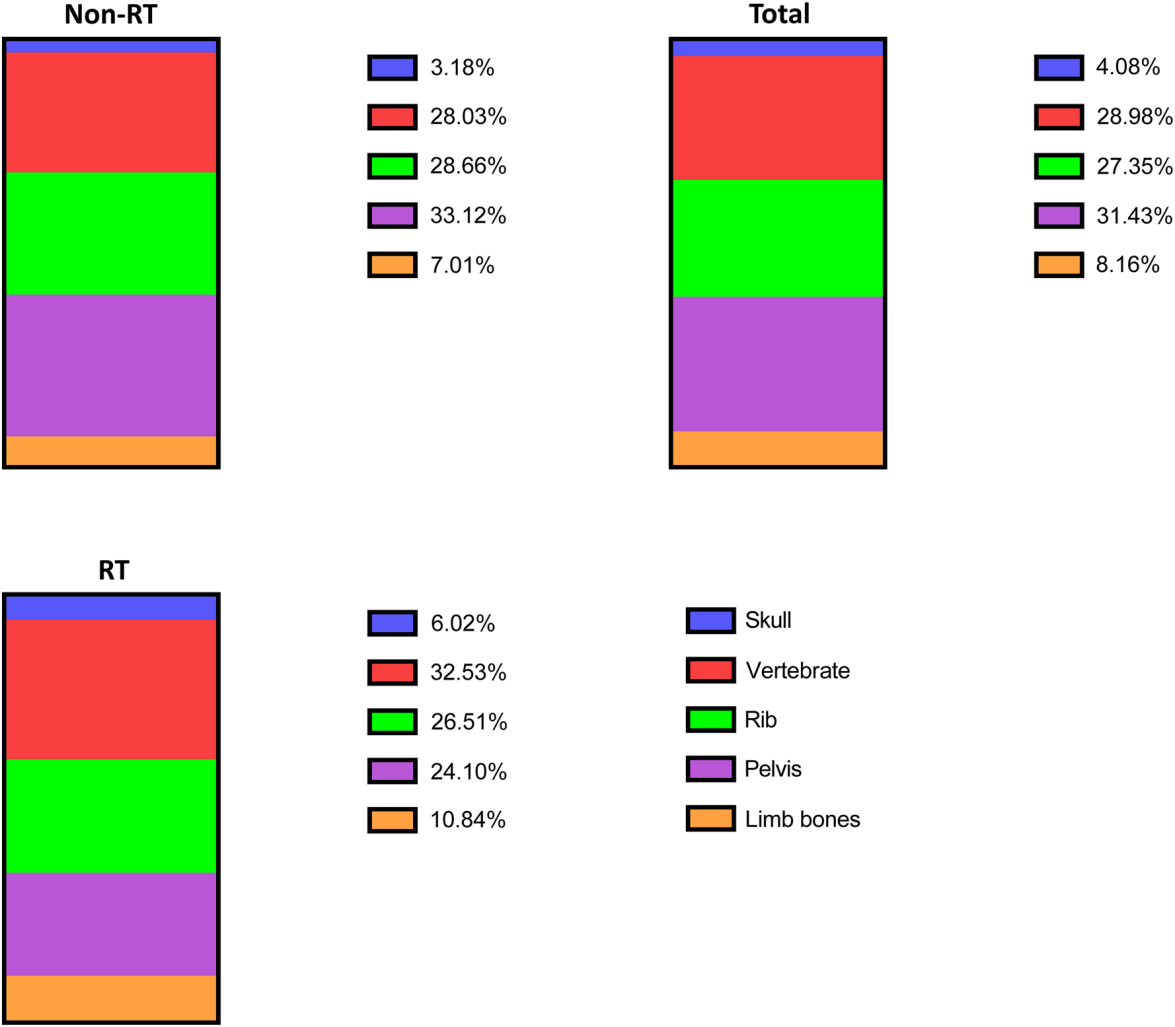
Sites of bone metastases from PCa in RT and non-RT group. *PCa* prostate cancer; *RT* radiotherapy

Vertebrae, ribs and pelvises were the most common metastasis sites in PCa patients. Figure 1 shows bone metastatic sites in patients.

### Survival rate analysis

**Fig. 2:**
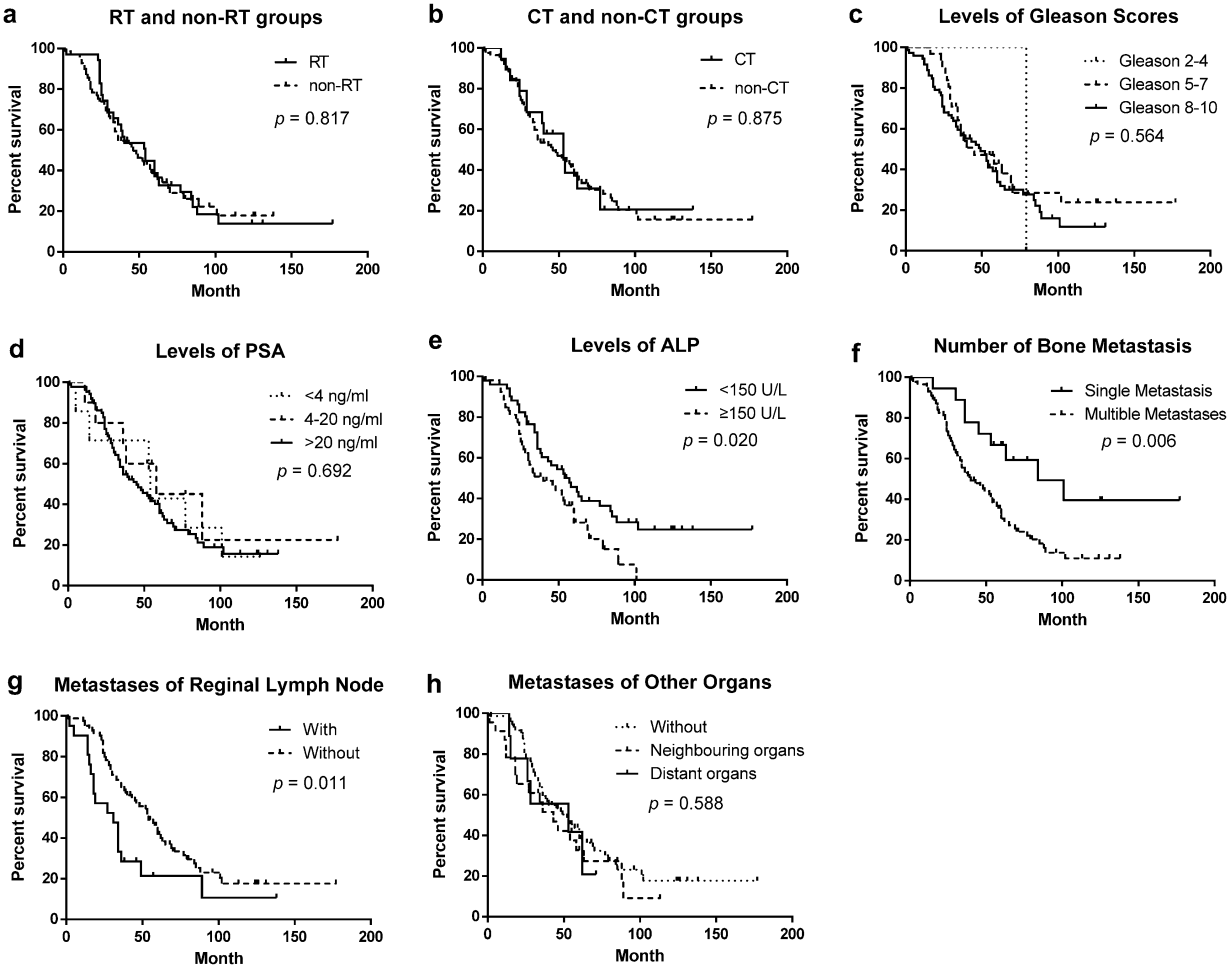
The overall survival curves of patients with Kaplan–Meier estimator, tested with a log-rank test. **a** The overall survival curves of RT and non-RT groups, *p* = 0.817. **b** The overall survival curves of CT and non-CT groups, *p* = 0.875. **c** The overall survival curves of patients with different levels of Gleason scores, *p* = 0.564. **d** The overall survival curves of patients with different levels of PSA, *p* = 0.692. **e** The overall survival curves of patients with different levels of ALP, *p* = 0.020. **f** The overall survival curves of patients with single or multiple bone metastases, *p* = 0.006. **g** The overall survival curves of patients with or without metastases of reginal lymph nodes, *p* = 0.011. **h** The overall survival curves of patients with or without metastases of other organs, *p* = 0.588. *RT* radiotherapy; *CT* chemotherapy; *PSA* prostate-specific antigen; *ALP* alkaline phosphatase

From the diagnosis of bone metastases, the 1-, 2-, 3-, 5-, 7- and 10-year survival rates were 97.14, 82.86, 62.61, 38.76, 25.83 and 13.84 % respectively in the RT group, and 92.75, 73.91, 54.66, 36.63, 26.03 and 17.85 % respectively in the non-RT group. The survival rate of RT and non-RT group showed no significant difference (Table 2 and Fig. 2).

**Table 2:** Univariate analysis for survival

Factors	N	Survival rate (%)						Mean survival (month)	Median survival (month)	*P* value
		1 year	2 years	3 years	5 years	7 years	10 years			
Radiotherapy on metastasis sites										0.817
With	35	97.14	82.86	62.61	38.76	25.83	13.84	66.1	54	
Without	69	92.75	73.91	54.66	36.63	26.03	17.85	59.4	46	
Chemotherapy										0.875
With	19	94.74	78.95	68.42	38.6	20.58	20.58	62.3	53	
Without	85	94.12	76.47	54.76	36.82	26.48	15.61	65.2	46	
Gleason scores when diagnosed as PCa										0.564
2–4	1	100.00	100.00	100.00	100.00	0.00	0.00	79.0	79	
5–7	31	100.00	90.32	57.45	42.79	28.53	23.77	75.5	45	
8–10	72	91.67	70.83	56.66	33.80	24.96	11.88	55.0	49	
PSA when bone metastases occurred										0.692
<4 ng ml^−1^	7	85.71	71.43	71.43	42.86	28.57	14.29	61.4	54	
4–20 ng ml^−1^	10	90.00	80.00	70.00	45.00	45.00	22.50	78.6	58	
>20 ng ml^−1^	87	95.40	77.01	54.71	35.74	23.36	15.73	58.1	45	
ALP when bone metastases occurred										0.020
<150 U I^−1^	51	96.09	82.35	64.4	45.6	33.74	24.78	78.6	57	
≥150 U I^−1^	53	82.45	71.70	50.8	28.16	15.08	0.00	46.7	40	
The number of bone metastases										0.006
Single	18	100	94.44	77.78	66.67	49.38	39.51	104.8	84	
Multiple	86	93.02	73.26	53.07	30.46	20.35	10.97	53.1	40	
Regional lymph node metastases										0.011
With	21	90.48	57.14	28.57	21.43	21.43	10.71	71.0	54	
Without	83	95.18	81.93	64.82	41.60	27.51	17.59	42.9	31	
Metastases when bone metastases occurred										0.588
Without	72	98.61	80.56	59.35	38.29	25.54	17.69	69.5	52	
Neighbouring organs^a^	23	78.26	65.22	51.51	32.78	27.31	–	47.9	43	
Distant organs^b^	9	100.00	77.78	55.56	41.67	–	–	44.3	53	

*ALP* alkaline phosphatase; *PSA* prostate-specific antigen

^a^ Defined as an organ around with prostate, such as the bladder, spermatophores, urethra and rectum

^b^ Defined as brain, lung and liver metastases

As the significant difference of regional lymph node metastasis between RT and non-RT group, stratified analysis was performed. When adjusted for this factor, there was still no significant difference between the RT and non-RT groups (Table 3).

**Table 3:** Stratified analysis of radiotherapy

Regional lymph node metastases	N	Mean survival (month)	Median survival (month)	Adjusted *p* value
With				
RT	1	2.0	2	0.596
Non-RT	20	45.0	31	
Without				
RT	34	68.0	54	
Non-RT	49	63.5	57	

### Prognostic factor analysis

The univariate analysis of the results indicated that the survival was associated with ALP when bone metastases occurred, the number of bone metastases, and regional lymph node metastases when bone metastases occurred (*p* < 0.05). The differences for other factors were not statistically significant (*p* > 0.05, Table 2 and Fig. 2).

Multivariate Cox regression analysis indicated that ALP when bone metastases occurred and the number of bone metastases were significant factors of survival, while the radiotherapy was not. The coefficient of regression and relative risk were showed in Table 4.

**Table 4:** Multivariate analysis for survival

Factors	β	S.e.	RR	*P* value
Radiotherapy on metastasis sites	0.209	0.289	1.233	0.469
Chemotherapy	−0.198	0.316	0.820	0.530
Gleason scores when diagnosed as Pca^a^				0.876
2–4	−0.507	1.037	0.602	0.625
5–7	−0.054	0.277	0.948	0.846
PSA when bone metastases occurred^b^				0.569
<4 ng ml^−1^	0.492	0.475	1.635	0.301
4–20 ng ml^−1^	0.214	0.489	1.239	0.661
ALP when bone metastases occurred	0.461	0.251	1.586	0.041
The number of bone metastases	0.889	0.410	2.433	0.030
Regional lymph node metastases	0.580	0.321	1.786	0.071
Metastases when bone metastases occurred^c^				0.957
Without	−0.108	0.443	0.897	0.807
Neighbouring organs	−0.050	0.511	0.951	0.922

*ALP* alkaline phosphatase; *β* coefficient of regression; *PCa* prostate cancer; *RR* relative risk; *s.e.* standard error

^a^ Gleason 8–10 used as control group

^b^ PSA > 20 ng ml-1 used as control group

^c^ Distant organs used as control group

## Discussion

The clinical features of bone metastases from PCa are similar with other malignancies in which bone metastases most commonly affect the axial skeleton [7]. The most common metastatic sites are the vertebrae, ribs and pelvis. RT is one of the most important therapeutic options available in advanced PCa stage. Its effects have been recognized for local control of bone metastases and reducing the SREs. However, when it comes to the long-term prognosis of RT for PCa bone metastases, it is still controversial [8–11].

Conventionally, RT is just a palliative treatment for advanced PCa patients with much lower doses to destroy the tumor cells. The exact mechanism of palliative RT has not been well defined, which may be the effects on bone homeostasis or alteration of signaling pathways [8, 12]. Comparing with the radical prostatectomy, which dose is recommended for greater than 70 Gy, the procedure of palliative RT is commonly a single-fraction dose (4–8 Gy) or multi-fraction higher doses (20–45 Gy) [8–10, 13]. In our study, the full radiation dose for the patients is 46 Gy, and part of the patients had less dose due to side effects and other reasons. It is demonstrated that the dosage and schedule of RT is associated with the overall survival of PCa patients with bone metastasis. Tabata et al. [11] had shown that the oligometastases and oligo recurrence of bone metastases in PCa patients treated with conventional RT doses of >40 Gy had a superior 3-year survival compared with those treated with <40 Gy (90.5 vs 50.0 %, *p* = 0.012). Wu et al. had also indicated that the long-course RT (37.5 Gy in 15 fractions, 40 Gy in 20 fractions, or 50 Gy in 25 fractions) had a better 3-year overall survival than short-course RT (20 Gy in 5 fractions or 30 Gy in 10 fractions) in bone oligometastases PCa patients underwent RT combined with endocrine therapy after curative RT for PCa (76.4 vs 44.1 %, *p* = 0.03) [14]. In our study, that the survival between RT and non-RT group showed no significant difference may be the result of insufficient dose of RT.

Another critical factor that influences the result is the sites for RT. As mentioned in methods, for patients with multiple bone metastases, those with lesions causing pain or possibly spinal cord compression were first considered for RT. Not all lesions received radiation so that it might influence the long-term survival. To have better effect on the diffuse lesions, radionuclides were introduced for the treatment to bone metastatic cancer. Several radionuclides have shown effects on the relief of SREs, like strontium-89, samarium-153 and radium-223. Among them, radium-223 is the most promising radionuclide that could improve the survival [15]. Radium-223 is a bone-seeking calcium mimetic, which selectively binds to areas of increased bone turnover in bone metastases, and emits high-energy alpha particles of short range (<100 µm) [16]. A phase 3, randomized, double-blind, placebo-controlled study conducted by Parker et al. [17] showed that radium-223 significantly improved overall survival of bone metastatic PCa patients compared with placebo (14.0 months vs. 11.2 months; HR 0.70; 95 % CI 0.55–0.88; *p* = 0.002). The study was terminated for efficacy at the interim analysis and the result was published on the NEJM.

In our study, the number of bone metastases, the ALP when bone metastases occurred and regional lymph node metastases were the prognostic factors analyzed by univariate analysis. When adjusting for confounding variables, only the number of bone metastases and the ALP when bone metastases occurred had significant difference. The number of bone metastases had a strong impact on the survival, which was in agreement with previous studies. Singh et al. [18] reported that patients with five or less metastatic lesions had higher 5-year overall survival compared with those with five lesions (73 vs 45 %), which was similar to metastasis-free patients. Schick et al. reported a 3-year biochemical recurrence-free survival of 66.5 % in patients treated with androgen deprivation combined with high-dose RT for only one metastatic lesion and 36.4 % (*p* = 0.031) in those treated for more than one metastases [19]. Similarly, another study by Wu et al. [14] showed that significantly improved three-year overall survival was observed in PCa patients with one metastatic lesion compared with patients with more than one metastatic lesions (78.8 vs 42.2 %). For patients with single or limited bone metastases, they may get better prognosis with aggressive RT. Therefore, Hellman and Weichselbaum firstly hypothesized that the local treatment of patients with limited number of metastatic or recurrent lesions, using surgical resection and RT, improved systemic control. In this situation, the number of metastases was less than five, and the primary lesion could be controlled [20]. It further arose the viewpoint for restaging the stage IV cancer. Rubin suggested amending the TNM staging system, modifying the “M” to represent solitary metastasis (M1), oligometastases (M2), or multiple metastases (M3) [21]. For oligometastases of PCa, it means a controlled or controllable primary lesion with five or fewer metastases (ideally 1–3) located in the bone (preferably the spine, or ganglions) [22]. With a few retrospective studies, it was suggested that local therapy to a small number of gross metastatic sites and recurrences might result in prolonged survival or even cure [14, 19, 20, 22, 23]. Several phase II prospective clinic trials are seeking for more convincing evidence for the treatment to oligometastases of PCa [22, 24].

With the advances in the treatment of metastatic PCa, the viewpoint has changed from palliative treatment to life-prolonging treatment. Several agents have shown effects on the improvement of survival for metastatic PCa patients, including docetaxel with prednisone, cabazitaxel with prednisone, Sipuleucel-T, abiraterone with prednisone, enzalutamide and radium-223 [25]. In order to prolong the survival of the metastatic PCa patients, radiotherapy may not be the first choice. However, for palliative treatment only, single fraction of RT is still considered as an effective and cost-effective method [5, 26, 27]. Therefore, the choice of treatment strategy should be considered thoroughly based on the state of the patients and the objective of the treatment.

## Conclusion

In conclusion, RT on the bone metastases cannot prolong the overall survival of young PCa patients. The number of bone metastases and the ALP when bone metastases occurred are the prognostic factors. In order to improve the survival of metastatic PCa patients, RT may not be the first choice.
